# Is overexpression of TWIST, a transcriptional factor, a prognostic biomarker of head and neck carcinoma? Evidence from fifteen studies

**DOI:** 10.1038/srep18073

**Published:** 2015-12-10

**Authors:** Xianlu Zhuo, Huanli Luo, Aoshuang Chang, Dairong Li, Houyu Zhao, Qi Zhou

**Affiliations:** 1 Post-doctoral scientific research station, Chongqing Cancer Institute, Chongqing, China; 2 Affiliated Hospital of Guiyang Medical University, Guiyang, China; 3 Department of Medical Oncology, Chongqing Cancer Institute, Chongqing, China; 4 Department of Gynecologic Oncology, Chongqing Cancer Institute, Chongqing, China

## Abstract

TWIST, a basic helix-loop-helix transcription factor, has been indicated to play a critical role in the progression of numerous malignant disorders. Published data on the significance of TWIST expression in head and neck carcinoma (HNC) risk have yielded conflicting results. Thus, we conducted a quantitative meta-analysis to obtain a precise estimate of this subject. After systematic searching and screening, a total of fifteen studies using immunohistochemistry for TWIST detection were included. The results showed that TWIST positive expression rate in HNC tissues was higher than that in normal tissues. TWIST expression might have a correlation with clinical features such as low differentiation, advanced clinical stage, presence of lymph node metastasis, distant metastasis and local recurrence (P < 0.05) , but not with age, gender, T stage and smoking as well as drinking (P > 0.05). In addition, over-expression of TWIST was a prognostic factor for HNC (HR = 1.92, 95% CI = 1.13–3.25). The data suggested that TWIST might play critical roles in cancer progression and act as a prognostic factor for HNC patients.

Head and neck carcinoma (HNC), the sixth most frequent kind of cancer worldwide, is a group of biologically similar cancers that originate from head and neck regions such as oral cavity, pharyngeal cavity, and larynx^1^. Previous reports showed that life-style factors such as smoking, drinking, betel quid chewing, papilloma virus infection, and exposure to toxic substances are possible etiological risk factors for HNC^2^,^3^. Besides, genetic variations might also play important roles in its genesis^4^. Hence, the etiological factors for this type of cancer are complicated. To find new biomarkers for predicting the prognosis of HNC patients is required.

Epithelial-mesenchymal transition (EMT) is a indispensable event for the formation of various organs during the process of embryonic development, whereas it may be suppressed for maintaining epithelial integrity in mature tissue^5^. Abnormal activation of EMT in epithelial tumors usually has been indicated to have a relationship with the genesis and development of a variety of cancers^6^.

Evidence shows that several transcriptional factors might act as inducers of EMT and thus play critical roles in its process. A basic helix-loop-helix (BHLH) transcription factor, TWIST, is one of the important EMT inducers. Reports showed that over-expression of TWIST might be associated with lymph node metastasis of thyroid cancer^7^ and gastric cancer^8^. In addition, TWIST act as a useful predictor of unfavorable prognosis for ovarian^9^ and renal cell carcinoma^10^. Hence, TWIST is involved not only in early events of malignancies, but contributes to cancer progression as well. Therefore, TWIST has been suggested as a potential target for cancer biotherapy and an important biomarker for predicting the prognosis of cancers^11^.

Previously, a growing body of studies has been conducted on the expression and significance of TWIST in HNC. However, the results were inconsistent. Since a single study was underpowered in demonstrating the roles of TWIST in HNC progression, we aimed to conduct a quantitative meta-analysis containing published data up to Jun 2015 that increased statistical power to get a more precise estimation. Since both TWIST1 and TWIST2 belong to the basic helix-loop-helix (bHLH) transcriptional factor family, and they share more than 90% sequence homology and structural similarity at bHLH and C-teminal domains and biological similarity in disorders^12^, studies on TWIST, TWIST1 and TWIST2 were all considered in the present study.

## Materials and Methods

### Literature search strategy

An internet literature search was carried out in the databases such as Medline, Ovid, Springer, EMBASE, and China National Knowledge Infrastructure (CNKI) without a language limitation, covering all papers published up to Jun 2015. A combination of the following keywords was used: *TWIST*, *EMT*, *head and neck neoplasm, tumor, cancer, pharynx, larynx, and mouth*. All searched studies were retrieved and the bibliographies were checked for other possible publications. Potential related review articles were hand searched to find additional eligible studies whenever necessary.

### Inclusion criteria

Several criteria were used for the literature selection: first, studies must concern the roles of TWIST, TWIST1 or TWIST2 expression in primary HNC tissues and assess its relationship with pathological features and Immunohistochemistry (IHC) was used as the major method for detection of TWIST expression; second, papers should provide clinical data of cancer cases who were not subjected to radiotherapy or chemotherapy prior to the investigation; third, studies must be observationally designed. Accordingly, the exclusion criteria were used as follows: first, the judgment standard for positive TWIST expression was obviously different from other papers; second, TWIST was detected from the blood circulation of patients, or studies only concerned animal experiments or cell line cultures; third, reviews, duplicate publications, or papers presented insufficient information from which we could not infer the results.

### Data extraction

Valuable information was carefully extracted from all eligible publications independently by two of the authors according to the inclusion criteria and illustrated in a database. For discrepancies of the data, a discussion was made to reach an agreement in case of conflicting evaluations. If a consensus were not reached, another author joined in to resolve the dispute and then a final decision was made by the majority of the votes.

### Statistical analysis

The pooled odd ratio (OR) and their 95% confidence interval (CI) was utilized to assess the relationship between TWIST expression and the clinicopathologic characteristics. Hazard ratio (HR) and its 95% CIs were used to evaluate the correlation between TWIST expression and the prognosis of patients with HNC, with its value of greater than 1 indicating poor outcome. HRs were directly extracted from the literature, estimated by the available information or estimated from the Kaplan-Meier curves according to the method raised by Tierney *et al.*^13^ if they were not directly reported in the primary literature. Between-study heterogeneity was assessed by a chi-square based Q statistic test. If a *P value* for a given Q-test was found to be more than 0.1, ORs were pooled according to a fixed-effect model (Mantel-Haenszel)^14^; otherwise, a random-effect model (DerSimonian and laird) was used^15^. Funnel plots^16^ were created to show the publication bias and a visually asymmetrical plot indicated a potential publication bias. The symmetry of the funnel plot was further determined by Egger’s linear regression test^17^. All statistical analysis was carried out by using the program STATA 11.0 software (Stata Corporation, Texas, USA).

## Results

### Study characteristics

**Figure 1:**
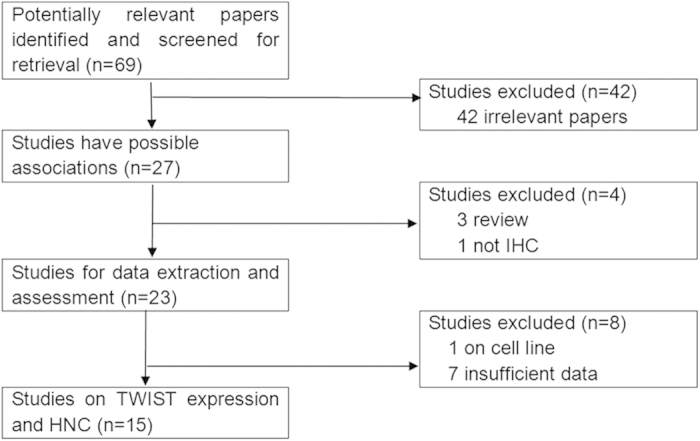
The flow diagram of included/excluded studies.

After a systematic search and screen, a total of sixty-nine publications were originally obtained, of which forty-two irrelevant papers were excluded. Thus, twenty-seven publications were eligible. Then, three review articles^18^,^19^,^20^ and one study in which IHC was not used^21^ were discarded. Next, one study that concerned cell line rather than tissue^22^ and seven papers that provided insufficient information^23^,^24^,^25^,^26^,^27^,^28^,^29^ were further excluded. Lastly, fifteen studies were selected for data extraction and evaluation^30^,^31^,^32^,^33^,^34^,^35^,^36^,^37^,^38^,^39^,^40^,^41^,^42^,^43^,^44^ (Fig. 1).

Among the included studies, nine were written in English^30^,^31^,^32^,^33^,^34^,^35^,^37^,^38^,^39^, while the remaining six were in Chinese^36^,^40^,^41^,^42^,^43^,^44^. The relevant information was listed in Table 1. According to this table, the first author and the number and characteristics of cases for each study as well as other necessary information were presented. Notably, only six papers reported the prognostic data, of which information about HR were directly extracted from three studies^31^,^32^,^38^, while in the remaining three studies^30^,^37^,^39^, HRs were indirectly estimated from the Kaplan-Meier curves according to the method reported by Tierney *et al.*^13^. In a study by Qian *et al.*^35^, though HR value was reported, its relevant 95% CI and Kaplan-Meier curves were absent. Thus, the information about HR value in this paper was discarded.

### Meta-analysis results

The main results of the present meta-analysis were presented in Table 2. For the overall data, the P value for the Q-test was 0.261, and thus, the between-study heterogeneity was insignificant and a fixed-effect model was selected for data pooling. However, heterogeneity could be shown in the subgroups regarding T stage, clinical stage, differentiation and lymph node metastasis, respectively. Therefore, random-effect models were used in these subgroups.

**Figure 2:**
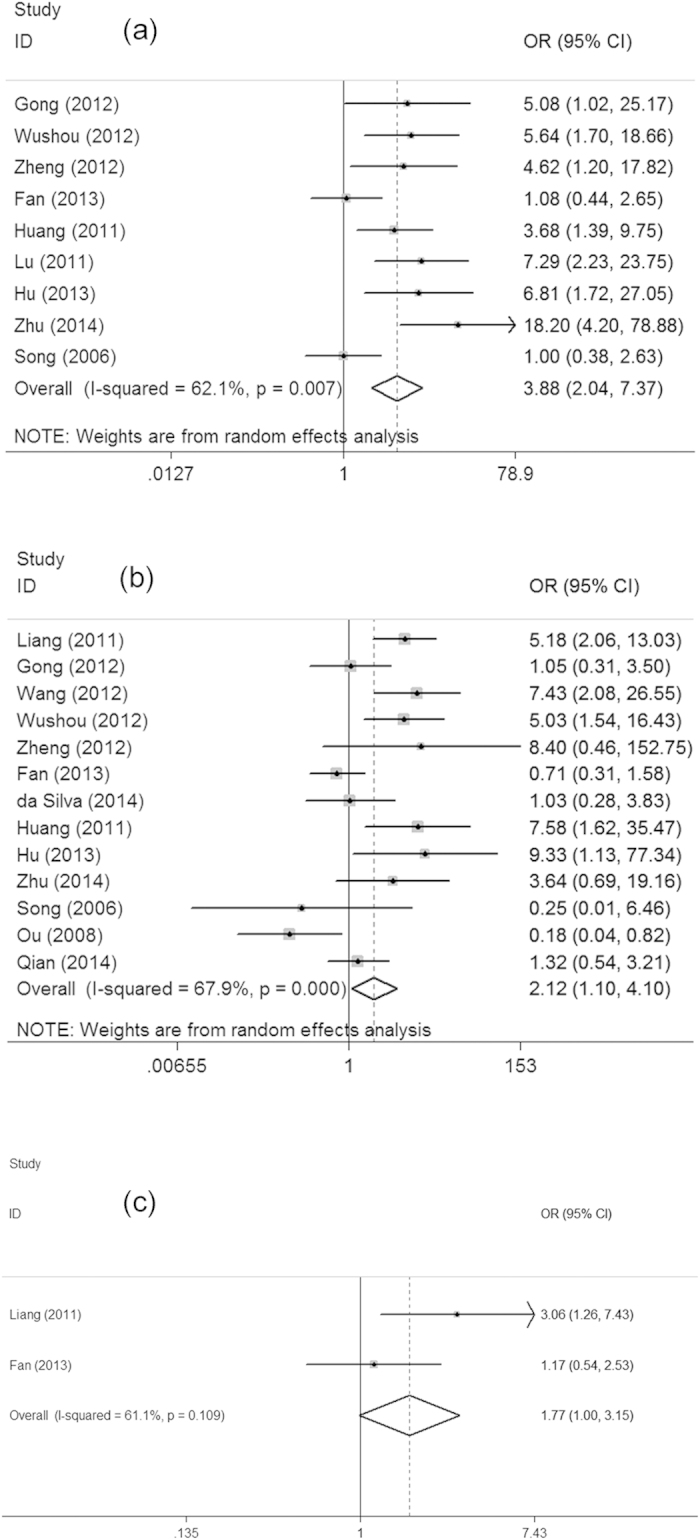
Forest plots showed that TWIST over-expression was associated with clinical stage (**a**). differentiation (**b**), local recurrence (**c**).

**Figure 3:**
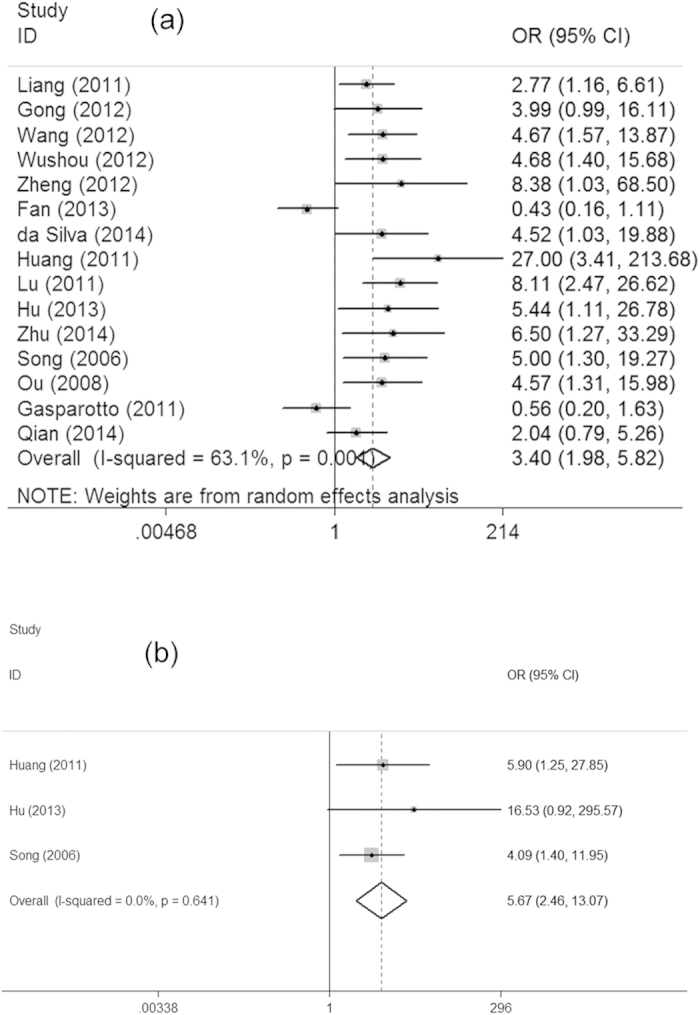
Forest plots showed that TWIST over-expression was associated with lymph node metastasis (**a**). and distant metastasis (**b**).

Positive expression of TWIST in HNC tissues were significantly higher than that in normal tissues (OR = 14.27, 95% CI = 8.22−24.79). As shown in this table, no association was found between TWIST expression and several clinicophathological features, such as age, gender, smoking, drinking and T stage. However, as shown in Fig. 2, TWIST over-expression was correlated with clinical stage (III + IV vs I + II, OR = 3.88, 95% CI = 2.04–7.37), differentiation (Low vs Moderate + High, OR = 2.12, 95% CI = 1.10–4.10) and local recurrence (Yes vs No, OR = 1.77, 95% CI = 1.00–3.15), respectively, indicating that TWIST might have an association with advanced stages of HNC. In addition, TWIST over-expression has a correlation with lymph node metastasis (Yes vs No, OR = 3.40, 95% CI = 1.98–5.82) and distant metastasis (Yes vs No, OR = 5.67, 95% CI = 2.46–13.07), suggesting that TWIST might contribute to cancer development and progression (Fig. 3).

**Figure 4:**
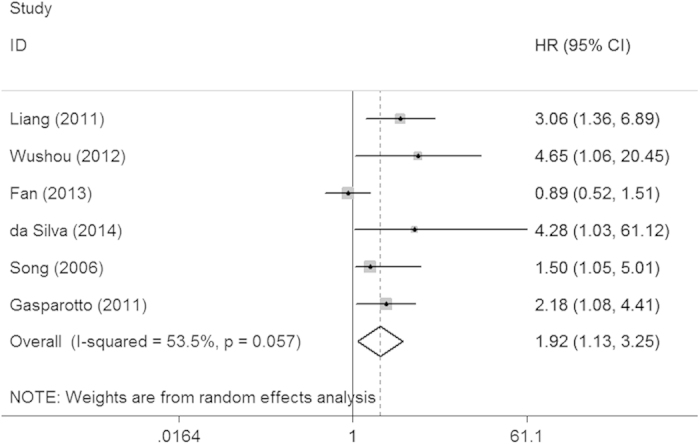
Forest plots showed that TWIST over-expression indicate a poor prognosis of patients with HNC.

To evaluate the prognostic value of TWIST for HNC, HRs for the overall survival were pooled. As shown in Table 2, the pooled HR was 1.92 (95% CI = 1.13–3.25), suggesting that over-expression of TWIST was a prognostic factor for HNC (Fig. 4).

To determine the stability of the above comparisons, one-way sensitivity analysis^45^ was performed in the comparisons, respectively. Consequently, the statistical significance of the results was not changed when any one study was deselected (data not shown) in the repeated analysis, indicating the robustness of the results.

### Bias diagnostics

**Figure 5:**
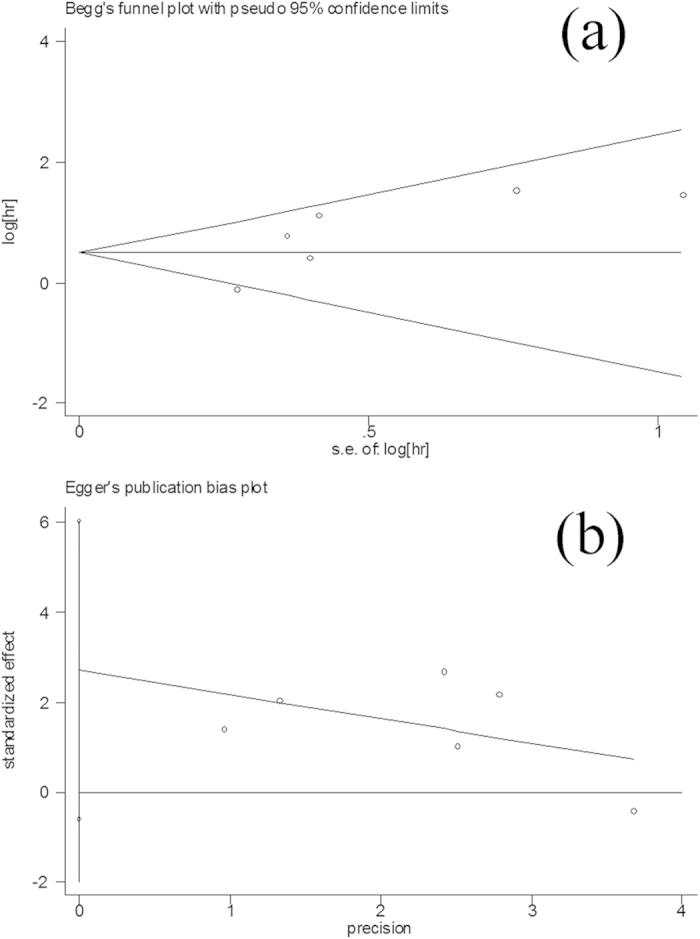
Publication bias tests for the pooled HR evaluation (TWIST positive vs TWIST negative). (**a**) Funnel plot; (**b**) Egger’s linear regression test.

Funnel plots were created to detect the possible publication bias. Then, Egger’s linear regression tests were used to assess the symmetries of the plots. The results showed that the publication bias was not significant for the pooled HRs comparison (t = 2.27, P > 0.05), indicating little effect of the publication bias on the overall results (Fig. 5).

## Discussion

In the present meta-analysis, the pooled results from fifteen primary studies showed that TWIST expression might have an association with low differentiation, advanced clinical stage, presence of lymph node metastasis, distant metastasis and local recurrence, indicating that TWIST expression might play an important role in the development of HNC. In addition, TWIST might act as a prognostic factor for HNC.

HNC can severely affect the psychological health and life quality of patients because this type of cancer may directly influence speaking, eating and breathing due to its specific site. The underlying mechanisms of HNC development are not fully understood. Recently, much attention has been focused on EMT because it is representative of a transition in which cells lose their epithelial polarity and gain mesenchymal properties with increased mobility^46^. Based on this alteration, cancer cells become more malignant and have the tendency of being aggressive. The EMT process can be induced by a number of cytokines such as TGF-beta^47^, CTGF^48^ and HIF-1α, particularly in hypoxic microenvironment that resulted from excessive growth of cells^49^. Thus, EMT acts as a key event in the development of cancers and the critical molecules or signaling pathways involved in EMT have been regarded as potential targets for tumor biotherapy^50^. TWIST is one of the important inducers of EMT process. Several published meta-analyses have concerned the relationship of TWIST expression with cancers. For example, TWIST expression is associated with poor prognosis in patients with lung cancer^51^ and oral cancer^52^. There were two meta-analyses published in 2014 have concerned the roles of TWIST in the prognosis of HNC and have generated conflicting results. One meta-analysis by Wushou *et al.*^53^ suggested that TWIST1 is a prognostic factor for HNC, with the value of the pooled HR 1.50 (95% CI:1.08–2.08). Nevertheless, the other one by Zhang *et al.*^54^ addressing the same issue showed that the HR for overall survival was 1.62 (95% CI: 0.78–3.38), indicating that TWIST may not be an unfavourable prognostic indicator in HNC. The discrepancy might be due to the reason that the criteria for literature inclusion were different, and the number of the included studies regarding HNC was limited (only three) for these two meta-analyses, respectively. Additionally, the relationship between TWIST expression and clinical features had not been assessed. Hence, compared with these two published papers^53^,^54^, the greater number of the included studies with larger sample sizes in the present meta-analysis might increase power to get a more confidential estimate.

The precise molecular mechanisms of TWIST in cancer progression have not been fully demonstrated. As a BHLH factor, TWIST can regulate gene transcription by recognizing a unique spatial configuration of E-boxes^55^. Also, it promotes alteration of cells from the epithelial physiology to the mesenchymal phenotype^56^, and promotes prolonged TGF-β1-induced G2 arrest of cells, limiting the abilities of cells to repair and regenerate^57^. Therefore, the cancer cells become more malignant than before. Moreover, TWIST expression is positively correlated with increased microvessel density in cancers^10^,^58^, indicating that TWIST can promote angiogenesis possibly through up-regulation of various biological factors such as MMP-2, MMP-9^59^, and VEGF^60^. Thus, neovascularization might obviously facilitate the migration of cancer cells. The above evidence might help explain the possible reasons why TWIST contributes to the cancer development such as lymph node and distant metastasis. However, future studies are warranted for clarifying the exact mechanisms because the published evidence is limited.

Evidence indicates that tobacco use has been shown to correlate with up-regulated TWIST expression^61^. Benzo(a)pyrene in tobacco might modulate TWIST expression and promote the migration and invasion of cancer cells^62^. Thus, the possible synergistic effects of smoking and TWIST expression are of great interest to investigators. In the present meta-analysis, four of the included studies assessed their association. Nevertheless, no associations were found in this comparison, possibly owing to the limited sample sizes. Moreover, the relationship between alcohol exposure and TWIST expression has also been evaluated. The data also failed to reveal a significant association between them. Thus, future studies considering smoking, drinking and other life-style factors are needed to explore the interactions of confounding factors with TWIST on cancer risk.

Survival information was available in six studies, and nevertheless, the HR values could be extracted directly from three papers^31^,^32^,^38^ and indirectly estimated from the Kaplan-Meier curves in another three papers^30^,^37^,^39^. The pooled HRs for the overall survival showed a significant difference between TWIST positive cases and negative cases, indicating that patients with positive or high TWIST expression had a worse prognosis compared with that of the ones with negative or low TWIST expression. However, the results should be interpreted with caution because any subjective errors might exist when interpreting the curves.

The roles of human papillomavirus (HPV) infection in the genesis of HNC have attracted much attention. HPV is a non-enveloped, double-stranded, epitheliotrophic, circular DNA virus that belongs to family Papovaviridae. Most cases of HPV positive HNC arise from the oropharyngeal region due to the possibility that this site is more vulnerable to epithelial injury and absence of protective keratin layer. HPV-positive HNCs have a favorable prognosis whereas HPV-negative ones exhibit a less favourable prognosis and a different molecular profile^63^, possibly because the former are primarily wild-type TP53, whereas HPV-negative tumors present mutated TP53 and show high chromosome instability^64^. Evidence indicates that HPV might also regulate TWIST expression and exert an effect on cancer progression^65^. However, the status of HPV infection has not been assessed in most of the included studies. Thus, the interaction of TWIST and HPV infection could not be determined in the present meta-analysis.

Several limitations might be included in this study. First, only published data in Chinese and English were involved. Papers included in other databases and published in other languages were ignored. Therefore, selection bias might exist. Second, the cut-off definition of TWIST appeared to be different in each study. This might affect the precision of the estimate. Third, the selected studies focused on TWIST expression in tissues rather than serum. Circulating prognostic markers have the tendency of being more convenient for detection. Fourth, heterogeneity might also be generated from the use of different anti-TWIST antibodies that were available from different companies, including rabbit or sheep polyclonal antibodies. This might exert an influence on the accuracy of TWIST detection. Fifth, most included studies in this meta-analysis concerned Chinese population and only a few concerned other ethnicities. Thus, the results might only be representative of a proportion of the people worldwide. Therefore, further well-designed investigations might be of value and interest for HNC research.

Despite the limitations, the data of the present meta-analysis showed a marked association of TWIST over-expression with low differentiation, advanced clinical stages, lymph node and distant metastasis as well as local recurrence, suggesting that TWIST might play critical roles in the development of HNC. In addition, TWIST over-expression might predict poor overall survival in patients with HNC. Future studies are needed to confirm the results.

## Additional Information

**How to cite this article**: Zhuo, X. *et al.* Is overexpression of TWIST, a transcriptional factor, a prognostic biomarker of head and neck carcinoma? Evidence from fifteen studies. *Sci. Rep.* **5**, 18073; doi: 10.1038/srep18073 (2015).

## Figures and Tables

**Table 1:** Characteristics of studies included in the present meta-analysis.

First Author	Year	Number of Patients			Cut-off of IHC	Method of quantification	Hazard ratio (95% CI)	site	Area	Outcome
		total	TWIST negative or low	TWIST positive or high						
Song	2006	75	42	33	≥ 5%	Percentage of staining	1.50 (1.05–5.01)*	Nasopharynx	China	(2) (3) (4) (5) (6) (11) (12)
Ou	2008	50	30	20	NA	NA	–	Combined	China	(1) (2) (3) (4) (5)
Gasparotto	2011	68	34	34	NA	NA	2.18 (1.08–4.41)	Combined	Italy	(5) (11)
Huang	2011	80	27	53	≥ 5%	Percentage of staining	–	Larynx	China	(1) (2) (4) (5) (6) (7) (12)
Liang	2011	89	49	40	≥ 5%	Percentage of staining	3.06 (1.36–6.89)*	Oral	China	(1) (2) (3) (4) (5) (8) (9) (10) (11)
Lu	2011	66	22	44	NA	Percentage of staining	–	Larynx	China	(1) (2) (5) (6)
Gong	2012	62	14	48	≥ 5%	Percentage of staining	–	Oral	China	(1) (2) (4) (5) (6) (7)
Wang	2012	60	30	30	≥ 4	Sum of percentage and intensity	–	Oral	China	(1) (2) (3) (4) (5)
Wushou	2012	60	18	42	≥ 3	Sum of percentage and intensity	4.65 (1.06–20.45)	Oral	China	(1) (2) (3) (4) (5) (6) (7) (8) (9) (11)
Zheng	2012	69	20	49	≥ 1	Sum of percentage and intensity	–	Oral	China	(1) (2) (4) (5) (6) (7)
Fan	2013	114	40	74	≥ 2	Sum of percentage and intensity	0.89 (0.52–1.51)	Oral	China	(1) (2) (3) (4) (5) (6) (7) (8) (9) (11)
Hu	2013	60	19	41	≥ 10%	Percentage of staining	–	Larynx	China	(1) (2) (4) (5) (6) (7) (12)
da Silva	2014	52	30	22	> 2	Extent of staining	4.28 (1.03–61.12)*	Oral	Brazil	(3) (4) (5) (10) (11)
Qian	2014	81	34	47	≥ 30%	Percentage of staining	–	Combined	Germany	(1) (2) (3) (4) (5) (8) (9)
Zhu	2014	49	19	30	4–12	Sum of percentage and intensity	–	Larynx	China	(1) (4) (5) (6) (7)

Clinical features: (1) Age; (2) Gender ; (3) T stage; (4) Differentiation; (5) Lymph node metastasis; (6) Clinical stage; (7) The control benign tissue; (8) Smoking; (9) Drinking. (10) Local recurrence; (11) Survival analysis (12) Distant metastasis.

*Estimated from the Kaplan-Meier curves in the text.

NA: Not available.

**Table 2:** Main results of the meta-analysis.

Clinical features	Overall OR (95% CI)	P	Heterogeneity test	P	Number of studies	Model
			Q			
TWIST expression (Cancer vs Normal)	14.27 (8.22–24.79)	< 0.05	7.70	0.261	7	Fixed-effect
Age (≥ 60 vs < 60)	0.98 (0.73–1.30)	> 0.05	12.19	0.350	12	Fixed-effect
Gender (Male vs Female)	1.09 (0.78–1.52)	> 0.05	6.78	0.817	12	Fixed-effect
T stage (T3 + T4 vs T1 + T2)	1.16 (0.65–1.95)	> 0.05	19.97	0.010	9	Random-effect
Differentiation (Low vs Moderate + High)	2.12 (1.10–4.10)	< 0.05	37.35	0.000	13	Random-effect
Lymph node metastasis (Yes vs No)	3.40 (1.98–5.82)	< 0.05	37.99	0.001	15	Random-effect
Clinical stage (III + IV vs I + II)	3.88 (2.04–7.37)	< 0.05	21.13	0.007	9	Random-effect
Local recurrence (Yes vs No)	1.77 (1.00–3.15)	< 0.05	2.57	0.109	2	Fixed-effect
Smoking (Yes vs No)	1.13 (0.70–1.81)	> 0.05	3.93	0.269	4	Fixed-effect
Drinking (Yes vs No)	0.84 (0.53–1.34)	> 0.05	0.88	0.830	4	Fixed-effect
Distant metastasis	5.67 (2.46–13.07)	< 0.05	0.89	0.641	3	Fixed-effect
Survival analysis	Overall HR (95% CI)	P	Heterogeneity test		Number of studies	Model
			Q	P		
TWIST ( + ) vs TWIST (−)	1.92 (1.13–3.25)	< 0.05	10.75	0.057	6	Random-effect
